# Generation of coherent spin-wave modes in yttrium iron garnet microdiscs by spin–orbit torque

**DOI:** 10.1038/ncomms10377

**Published:** 2016-01-27

**Authors:** M. Collet, X. de Milly, O. d'Allivy Kelly, V. V. Naletov, R. Bernard, P. Bortolotti, J. Ben Youssef, V. E. Demidov, S. O. Demokritov, J. L. Prieto, M. Muñoz, V. Cros, A. Anane, G. de Loubens, O. Klein

**Affiliations:** 1 Unité Mixte de Physique CNRS, Thales, Université Paris-Sud, Université Paris-Saclay, 1 avenue A. Fresnel, 91767 Palaiseau, France; 2 Service de Physique de l'État Condensé, CEA, CNRS, Université Paris-Saclay, CEA Saclay, Orme des Merisiers, 91191 Gif-sur-Yvette, France; 3 INAC-SPINTEC, CEA, CNRS and Université Grenoble Alpes, 17 avenue des Martyrs, 38000 Grenoble, France; 4 Institute of Physics, Kazan Federal University, Kazan 420008, Russian Federation; 5 Laboratoire de Magnétisme de Bretagne CNRS, Université de Bretagne Occidentale, 6 Avenue Le Gorgeu, 29285 Brest, France; 6 Department of Physics, University of Muenster, Correnstrasse 2-4, 48149 Muenster, Germany; 7 Institute of Metal Physics, Ural Division of RAS, Yekaterinburg 620041, Russian Federation; 8 Instituto de Sistemas Optoelectrónicos y Microtecnologa (UPM), Ciudad Universitaria, Madrid 28040, Spain; 9 IMM-Instituto de Microelectrónica de Madrid (CNM-CSIC), Isaac Newton 8, PTM, Tres Cantos, Madrid E-28760, Spain

## Abstract

In recent years, spin–orbit effects have been widely used to produce and detect spin currents in spintronic devices. The peculiar symmetry of the spin Hall effect allows creation of a spin accumulation at the interface between a metal with strong spin–orbit interaction and a magnetic insulator, which can lead to a net pure spin current flowing from the metal into the insulator. This spin current applies a torque on the magnetization, which can eventually be driven into steady motion. Tailoring this experiment on extended films has proven to be elusive, probably due to mode competition. This requires the reduction of both the thickness and lateral size to reach full damping compensation. Here we show clear evidence of coherent spin–orbit torque-induced auto-oscillation in micron-sized yttrium iron garnet discs of thickness 20 nm. Our results emphasize the key role of quasi-degenerate spin-wave modes, which increase the threshold current.

## Abstract (web summary)

Spin-orbit interactions allow for pure spin current to be injected into a ferromagnetic insulator from a current-carrying heavy metal, generating torque on the magnetization. Here, the authors evidence magnetic auto-oscillations driven by spin-orbit torque in thin film microdiscs of yttrium iron garnet.

## Graphical abstract

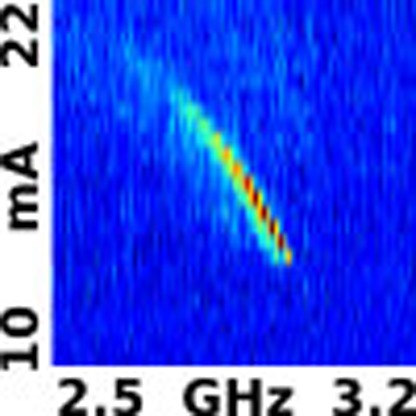

Spin–orbit effects^1^,^2^,^3^,^4^ have the potential of radically changing the field of spintronics by allowing transfer of spin angular momentum to a new class of materials. In a seminal letter to Nature, Kajiwara *et al*.^5^ showed that by depositing Platinum (Pt, a normal metal) on top of a 1.3-μm-thick yttrium iron garnet (YIG, a magnetic insulator), one could effectively transfer spin angular momentum through the interface between these materials. The outstanding feature was the detection of auto-oscillation of the YIG when enough d.c. current was passed in the Pt. This finding has created a great excitement in the community for two reasons: first, one could control electronically the damping of insulators, which can offer improved properties compared with metals, here YIG has the lowest damping known in nature; second, the damping compensation could be achieved on large objects, a relevant point for the field of magnonics^6^,^7^ whose aim is to use spin waves as carriers of information. However, the degree of coherence of the observed auto-oscillations was not addressed in ref. 5.

When spin transfer effects were introduced by Slonczweski and Berger in 1996 (refs 8, 9), the authors recognized that the striking signature of the process would be the emission of microwave radiation when the system is pumped out of equilibrium by a d.c. current. Since the spin transfer torque on the magnetization is collinear to the damping torque, an instability threshold occurs when the natural damping is fully compensated by the external flow of angular momentum, leading to spin-wave amplification through stimulated emission. Using analogy to light, the effect was called spin-wave amplification by stimulated emission of radiation (SWASER)^9^, where SW stands for spin wave. Until 2010, SWASER devices required a charge current perpendicular to the plane to transfer angular momentum between different magnetic layers^8^,^9^, which thus had to be conducting materials. The situation has changed since spin–orbit effects such as the spin Hall effect (SHE)^10^,^11^ are used to produce spin currents in normal metals. They allow the creation of a pure spin current transversely to the charge current, with an efficiency given by the spin Hall angle Θ_SH_. Using a metal with large Θ_SH_, such as Pt, a charge current flowing in plane generates a pure spin current flowing perpendicular to the plane, which can eventually be transferred through an interface with ferromagnetic metals, resulting in the coherent emission of spin waves^12^, but also with non-metals such as YIG^5^.

The microscopic mechanisms of transfer of angular momentum between a normal metal and a ferromagnetic layer are quite different depending on the latter being metallic or not. In the first case, electrons in each layer have the possibility to penetrate the other one, whereas in the second case the transfer takes place exactly and solely at the interface. It is thus much more sensitive to its imperfection. Still, a direct experimental evidence that spin current can indeed cross such a hybrid interface is through the spin-pumping effect^13^: adding a normal metal on top of YIG increases its ferromagnetic resonance (FMR) linewidth^14^, which is due to the new relaxation channel at the interface through which angular momentum can escape and get absorbed in the metal. This effect being interfacial, the broadening scales as 1/*t*_YIG_, where *t*_YIG_ is the thickness of YIG. Even for YIG, whose natural linewidth is only a few Oersted at 10 GHz, it is hardly observable if *t*_YIG_ exceeds a couple hundreds of nanometres. For these thick films though, the spin pumping can still be detected through inverse SHE (ISHE). In a normal metal with strong spin–orbit interaction, the pumped spin current is converted into a transverse charge current. This generates a voltage proportional to the length of the sample across the metal, which can reach several tens of microvolts in millimeter-sized samples. Since the first experiment by Kajiwara *et al*.^5^, many studies reported the ISHE detection of FMR using different metals on YIG layers^15^,^16^,^17^, hereby providing clear evidence of at least partial transparency of the hybrid YIG|metal interfaces to spin currents. Owing to Onsager relations, these results made the community confident that a spin current could thus be injected from metals to YIG and lead to the SWASER effect.

From the beginning it was anticipated that the key to observe auto-oscillations in non-metals was to reduce the threshold current. The first venue is to choose a material whose natural damping is very low. In this respect YIG is the optimal choice. The second thing is to reduce the thickness since the spin–orbit torque (SOT) is interfacial. This triggered an effort in the fabrication of ultra-thin films of YIG of high-dynamical quality^18^,^19^. For 20-nm-thick YIG films with damping constant as low as *α*=2.3 × 10^−4^, a striking result was that there were no evidence of auto-oscillations in millimeter-sized samples at the highest d.c. current possible in the top Pt layer^19^, that is, before it evaporates. It is worth mentioning that reducing further the thickness or the damping parameter of such ultra-thin YIG films^20^ does not help much in decreasing the threshold current, as the relevant value of the damping is that of the YIG|Pt hybrid, which ends up to be completely dominated by the spin pumping.

Also, most notably, none of these high-quality ultra-thin YIG films display a purely homogeneous FMR line^18^,^19^, for a well-known reason. In such extended films, there are many degenerate modes with the main, uniform FMR mode, which through the process of two-magnon scattering broaden the linewidth^21^,^22^. These modes can be revealed by parametric pumping^23^. Any threshold instability will be affected by their presence, as learnt from LASERs where mode competition is known to have a strong influence on the emission threshold^24^. Hence, the next natural step was to reduce the lateral size to lift the degeneracy between SW modes through confinement. The first microstructures of YIG revealed that patterning indeed narrows the linewidth through a decrease of the inhomogeneous part^25^. The effect is clear in the perpendicular geometry, where magnon–magnon processes are suppressed owing to the fact that the FMR mode lies at the bottom of the SW dispersion relation. This is not the case in the parallel geometry where the FMR mode is not the lowest energy mode. Even then, we showed that the linewidth in a micron-sized YIG|Pt disc can be largely reduced or enhanced through SOT^26^.

In the following, we describe the direct electrical detection of auto-oscillations in similar samples and show that the threshold current is increased by the presence of quasi-degenerate SW modes. This implies that careful engineering of the spin-wave mode spectrum is required to optimize magnonic devices making use of spin–orbit effects.

## Results

### Sample design

**Figure 1:**
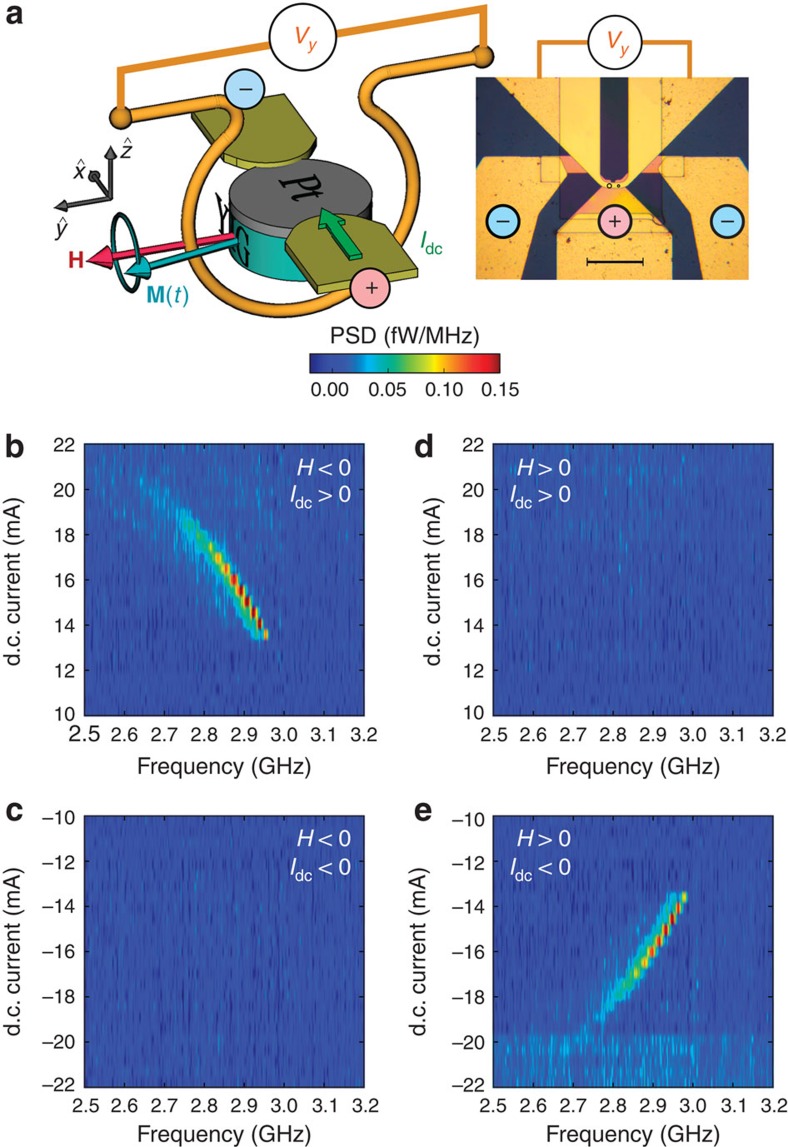
Inductive detection of auto-oscillations in a YIG|Pt microdisc. (**a**) Sketch of the measurement configuration and microscopy image of a device with two microdiscs connected (underneath the circles). Scale bar, 50 μm. The bias field **H** is oriented transversely to the d.c. current *I*_dc_ flowing in Pt. The inductive voltage *V*_*y*_ produced in the antenna by the precession of the YIG magnetization **M**(*t*) is amplified and monitored by a spectrum analyser. (**b–e**) PSD maps measured on a 4 μm YIG|Pt disc at fixed |*H*|=0.47 kOe and variable *I*_dc_. The four quadrants correspond to different possible polarities of *H* and *I*_dc_. An auto-oscillation signal is detected above a threshold current of ±13 mA if *H*·*I*_dc_<0, in agreement with the symmetry of spin–orbit torque.

We study magnetic microdiscs with diameter 2 and 4 μm, which are fabricated based on a hybrid YIG(20 nm)|Pt(8 nm) bilayer. The 20-nm-thick YIG layer is grown by pulsed laser deposition (PLD)^19^ and the 8-nm-thick Pt layer is sputtered on top of it^27^. Their physical parameters are summarized in Table 1. We stress that the extended YIG film is characterized by a low Gilbert damping parameter *α*_0_=(4.8±0.5) × 10^−4^ and a remarkably small inhomogeneous contribution to the linewidth^19^, Δ*H*_0_=1.9±0.5 Oe (full width at half maximum). Its magnetization 4*πM*_s_=2,150±50 G is ∼20% larger than the bulk value for YIG, which is attributed to a slight off-stoichiometry of the PLD grown material^19^. Each microdisc is connected to electrodes enabling the injection of a d.c. current *I*_dc_ in the Pt layer, and a microwave antenna is defined around it to obtain an inductive coupling with the YIG magnetization, as shown schematically in Fig. 1a.

### Detection of auto-oscillations

First, we monitor with a spectrum analyser the voltage produced in the antenna by potential auto-oscillations of the 4-μm YIG disc as a function of the d.c. current *I*_dc_ injected in Pt. The in-plane magnetic field **H** is applied in a transverse direction with respect to *I*_dc_, as shown in Fig. 1a. This is the most favourable configuration to compensate the damping and obtain auto-oscillations in YIG, as spins accumulated at the YIG|Pt interface due to SHE in Pt will be collinear to its magnetization. Colour plots of the inductive signal measured as a function of the relative polarities of **H** and *I*_dc_ are presented in Fig. 1b–e. The magnetic field is set to |*H*|=0.47 kOe. At *H*<0, we observe in the power spectral density (PSD) a peak which starts at around 2.95 GHz and 13 mA and then shifts towards lower frequency as *I*_dc_ is increased (Fig. 1b), a clear signature that spin transfer occurs through the YIG|Pt interface. An identical behaviour is observed at *H*>0 and *I*_dc_<0 (Fig. 1d). In contrast, the PSD remains flat in the two other cases (Fig. 1c–d). Therefore, an auto-oscillation signal is detected only if *H*·*I*_dc_<0, in agreement with the expected symmetry of SHE. Moreover, as opposed to numerous peaks previously observed over an almost 1 GHz wide frequency range^5^, a single emission peak is detected here by spectral analysis. The linewidth of this emission peak lies in the 10–20 MHz range for 13<|*I*_dc_|<17 mA. Depending on the bias conditions, we observe that the effective quality factor of the auto-oscillation signal, defined as the ratio of emission frequency to frequency linewidth, can reach values close to 1,000. This single mode with a narrow linewidth can hence be considered as coherent^28^.

### FMR spectroscopy

**Figure 2:**
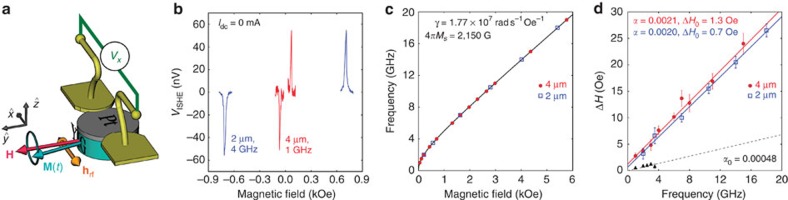
ISHE-detected FMR spectroscopy in YIG|Pt microdiscs. (**a**) Sketch of the sample and measurement configuration. The bias field **H** is oriented perpendicularly to the Pt electrode and to the excitation field **h**_rf_ produced by the antenna at fixed microwave frequency. The d.c. voltage *V*_*x*_ across Pt is monitored as a function of the magnetic field. (**b**) ISHE-detected FMR spectra of the 4- and 2-μm YIG(20 nm)|Pt(8 nm) discs at 1 and 4 GHz, respectively. (**c**) Dispersion relation of the main FMR mode of the microdiscs. The continuous line is a fit to the Kittel law. (**d**) Frequency dependence of the FMR linewidth in the two microdiscs. The continuous lines are linear fits to the data. The dashed line shows the homogeneous contribution of the bare YIG (the black triangles are the homogeneous contribution to the linewidth measured by standard FMR on the extended YIG film). The vertical bars show the mean squared error of the lorentzian fits.

To characterize the flow of angular momentum across the YIG|Pt interface, we now perform ISHE-detected FMR spectroscopy on our microdiscs. The configuration of this experiment is similar to the previous case, but now the antenna generates a uniform microwave field **h**_rf_ to excite the FMR of YIG while the d.c. voltage across Pt is monitored at zero current (Fig. 2a). In other words, we perform the reciprocal experiment of the one detailed before and presented in Fig. 1. As described in the introduction, a voltage *V*_ISHE_ develops across Pt when the FMR conditions are met in YIG. This voltage changes sign as the field is reversed, which is expected from the symmetry of ISHE, and shown in Fig. 2b, where the FMR spectra of the 4- and 2-μm microdiscs are, respectively, detected at 1 and 4 GHz. We also note that for a given field polarity, the product between *V*_ISHE_ and *I*_dc_ must be negative to compensate the damping^26^, which enables to observe auto-oscillations in Fig. 1.

From these ISHE measurements, the dispersion relation and frequency dependence of the full linewidth at half maximum of the main FMR mode can be determined, as shown in Fig. 2c,d, respectively. The dispersion relation follows the expected Kittel law. The damping parameters of the 4- and 2-μm microdiscs, extracted from linear fits to the data, Δ*H*=2*αω*/*γ*+Δ*H*_0_ (continuous lines in Fig. 2d, *ω* is the pulsation frequency and *γ* the gyromagnetic ratio), are found to be similar with an average value of *α*=(2.05±0.1) × 10^−3^. The small inhomogeneous contribution to the linewidth observed in both microdiscs, Δ*H*_0_=1.3±0.4 Oe and Δ*H*_0_=0.7±0.4 Oe, respectively, decreases with the diameter and is attributed to the presence of several unresolved modes within the resonance line^26^. To emphasize the increase of damping due to Pt, we have reported in Fig. 2d the broadening produced by the homogeneous contribution of the bare YIG using a dashed line. The observed increase of damping is due to spin pumping^13^,^14^,


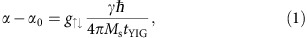


where *ħ* is the reduced Planck constant, *M*_s_ the saturation magnetization and *g*_↑↓_ the spin-mixing conductance of the YIG|Pt interface. This allows us to extract *g*_↑↓_=(3.6±0.5) × 10^18^ m^−2^, which lies in the same window as previously reported values^25^,^29^. From the spin-mixing conductance *g*_↑↓_, the spin diffusion length *λ*_SD_ and the resistivity *ρ* of the Pt layer, we can also calculate the transparency of the YIG|Pt interface to spin current^30^, *T*=0.2±0.05. The physical parameters extracted for the YIG|Pt hybrid bilayer are summarized in the last row of Table 1.

### Angle dependence

**Figure 3:**
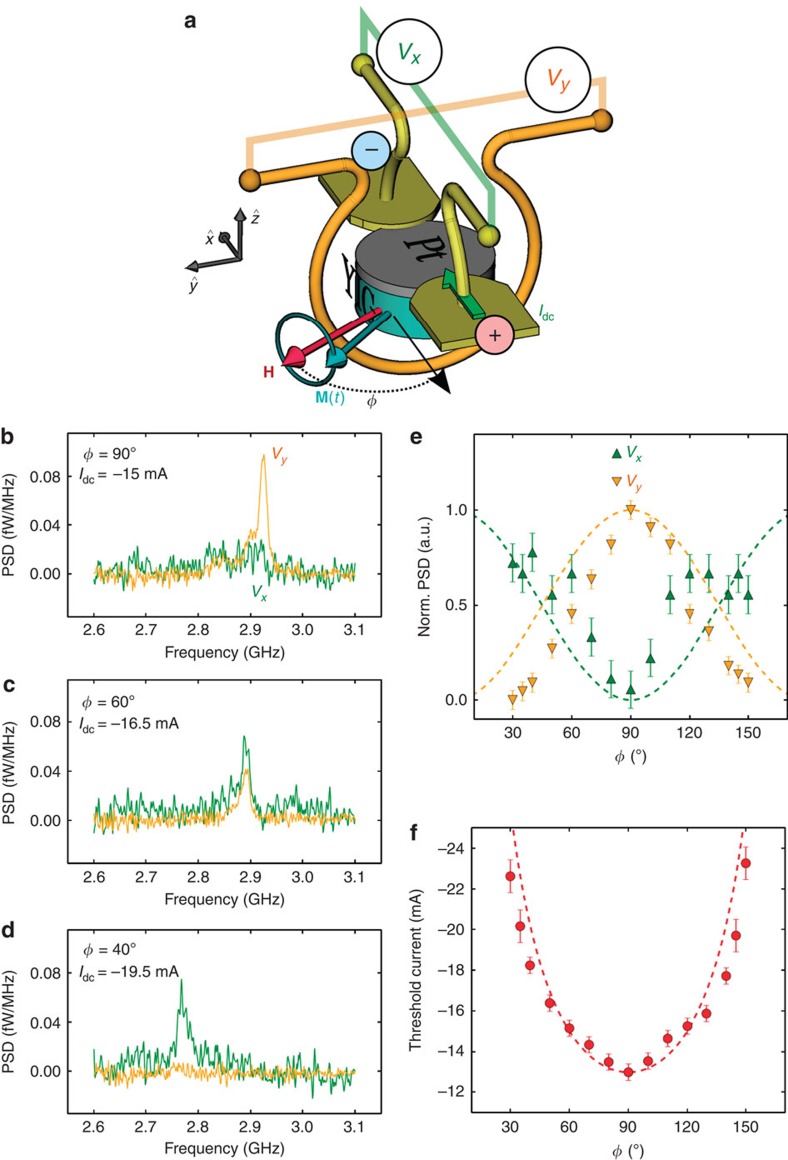
Auto-oscillations as a function of the angle between the d.c. current and the bias field. (**a**) Sketch of the sample and measurement configuration. The bias field **H** is oriented at an angle *φ* with the d.c. current *I*_dc_ in the Pt. The precession of the YIG magnetization induces voltages *V*_*x*_ in the antenna and *V*_*y*_ across Pt, which are amplified and monitored by spectrum analysers. (**b**–**d**) *V*_*x*_ and *V*_*y*_ at *H*=0.47 kOe for three different angles *φ* in the 4-μm disc. (**e**) Dependence of the normalized signals in both circuits and (**f**), of the threshold current for auto-oscillations on *φ*. In **e**,**f** dashed lines show the expected angular dependences and error bars are estimated from the limited signal-to-background contrast of the inductive signals.

To gain further insight about the origin of the auto-oscillation signal, we now monitor how the auto-oscillations of the 4-μm disc evolve as the angle *φ* between the in-plane bias field fixed to *H*=0.47 kOe and the d.c. current *I*_dc_ is varied from 30 to 150°. The results are summarized in Fig. 3. Pannels b–d show the auto-oscillation voltages detected in the antenna (*V*_*y*_) and across the Pt electrode (*V*_*x*_). At *φ*=90°, the auto-oscillation signal is only visible in the *V*_*y*_ channel. At *φ*=60°, both *V*_*x*_ and *V*_*y*_ channels exhibit the auto-oscillation peak. At *φ*=40°, it almost vanishes in *V*_*y*_, while it slightly increases in *V*_*x*_. The normalized signals as a function of *φ* are plotted in Fig. 3e. The Pt electrode and antenna loop being oriented perpendicularly to each other (Fig. 3a), the a.c. flux owing to the precession of magnetization picked up by each of them, respectively, varies as cos *φ* and sin *φ* (dashed lines in Fig. 3e).

More importantly, this study of angle dependence also allows us to extract the threshold current for auto-oscillations as a function of *φ*. As *φ* deviates from the optimal orientation 90°, the absolute value of the threshold current rapidly increases, see Fig. 3f. In fact, the SOT acting on the oscillating part **m** of the magnetization scales as **m** × **s** × **m**∝sin *φ*, where **s** is the spin polarization of the d.c. spin current produced by SHE in Pt at the YIG|Pt interface. Therefore, the expected threshold current scales as 1/sin *φ*, which is plotted as a dashed line in Fig. 3f, in very good agreement with the data.

In summary, the results reported in Figs 1 and 3 unambiguously demonstrate that the auto-oscillations observed in our hybrid YIG|Pt discs result from the action of SOT produced by *I*_dc_. We have also shown that they correspond to the reverse effect of the spin-pumping mechanism illustrated in Fig. 2d and its detection through ISHE in Fig. 2b.

### Quantitative analysis

**Figure 4:**
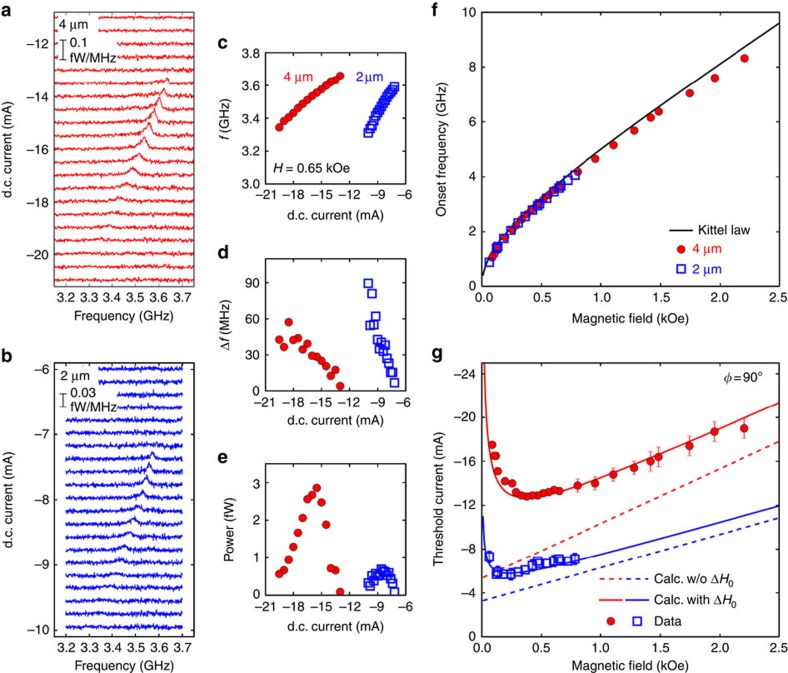
Quantitative analysis of auto-oscillations in YIG|Pt microdiscs. (**a**) Inductive voltage *V*_*y*_ produced by auto-oscillations in the 4-μm and (**b**) 2-μm YIG|Pt discs as a function of the d.c. current *I*_dc_ in the Pt. The experimental configuration is the same as in Fig. 1a, with the bias field fixed to *H*=0.65 kOe. (**c**) Auto-oscillation frequency *f*, (**d**) linewidth Δ*f* and (**e**) integrated power versus *I*_dc_. (**f**) Dependence of the onset frequency and (**g**) of the threshold current on the applied field in both discs. Expectations taking into account only the homogeneous linewidth or the total linewidth are respectively shown by dashed and continuous lines. Error bars are estimated from the limited signal-to-background contrast of the inductive voltage.

We now analyse quantitatively the main features of auto-oscillations, which allows us to determine their nature and to understand the role of quasi-degenerate SW modes in the SOT-driven dynamics. For this, we compare the auto-oscillations observed in the 4- and 2-μm microdiscs. Figure 4a,b, respectively, present the inductive signal *V*_*y*_ detected in the antenna coupled to these two discs as a function of *I*_dc_. The configuration is the same as depicted in Fig. 1a, with a slightly larger bias field set to *H*=0.65 kOe. One can clearly see a peak appearing in the PSD close to 3.6 GHz in both cases, at a threshold current of approximately −13.5 mA in the 4-μm disc and −7.4 mA in the 2-μm disc. These two values correspond to a similar threshold current density in both samples of (4.4±0.2) × 10^11^ A m^−2^, in agreement with our previous study^26^. As the d.c. current is varied towards more negative values, the peaks shift towards lower frequency (Fig. 4c), at a rate which is twice faster in the smaller disc. This frequency shift is mainly due to linear and quadratic contributions in *I*_dc_ of Oersted field and Joule heating^26^, respectively, (from the Pt resistance, the maximal temperature increase in both samples is estimated to be +40 °C). At the same time, the signal first rapidly increases in amplitude, reaches a maximum, and then, more surprisingly, drops until it cannot be detected anymore, as seen in Fig. 4e, which plots the integrated power versus *I*_dc_. The maximum of power measured in the 4-μm disc (2.9 fW) is four times larger than the one measured in the 2-μm disc (0.7 fW), which is due to the inductive origin of *V*_*y*_. The latter can be estimated from geometrical considerations, *V*_*y*_=*η*(*ωμ*_0_*Dt*_YIG_*M*_s_ sin *θ*)/2. Here *μ*_0_ is the magnetic constant, *D* the diameter of the disc and *θ* the angle of uniform precession (the prefactor *η* ≃0.1 accounts for microwave losses and impedance mismatch in the measured frequency range with our microwave circuit). For the same *θ*, the inductive voltage produced by the 4-μm disc is thus twice larger than by one produced by the 2-μm disc, hence the ratio four in power. Moreover, the maximal angle of precession reached by auto-oscillations is found to be ∼1° in both microdiscs^26^. Finally, the disappearance of the signal as *I*_dc_ gets more negative is accompanied by a continuous broadening of the linewidth, which increases from a few MHz to several tens of MHz (Fig. 4d). This rather large auto-oscillation linewidth is also consistent with a small precession angle, that is, a small stored energy in the YIG oscillator^31^.

By repeating the same analysis as a function of *H*, we can determine the bias field dependence of the auto-oscillations in both microdiscs. The onset frequency and threshold current at which auto-oscillations start are plotted in Fig. 4f,g, respectively. The onset frequency in the 4- and 2-μm microdiscs is identical and closely follows the dispersion relation of the main FMR mode plotted as a continuous line. The small redshift towards lower frequency, which increases with the applied field, is ascribed to the Joule heating and Oersted field induced by *I*_dc_ (the Kittel law in Figs 2c and 4f is obtained at *I*_dc_=0 mA). We also note that the main FMR mode is the one which couples most efficiently to our inductive electrical detection, because it is the most uniform. Hence, we conclude that the detected auto-oscillations are due to the destablization of this mode by SOT.

## Discussion

To reach auto-oscillations, the additional damping term due to SOT has to compensate the natural relaxation rate Γ_*r*_ in YIG. Given the transparency *T* of the YIG|Pt interface and the spin Hall angle Θ_SH_ in Pt, this condition writes^26^,^32^:


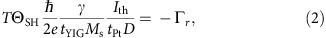


where *t*_Pt_*D* is the section of the Pt layer. The homogeneous contribution to Γ_*r*_ is given by the Gilbert damping rate, which for the in-plane geometry is^28^:


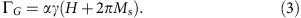


We remind that this expression is obtained by converting the field linewidth to frequency linewidth through Δ*ω*=Δ*H*(∂*ω*/∂*H*). If only the homogeneous contribution to the linewidth is taken into account, the threshold current *I*_th_ is thus expected to depend linearly on *H*, as shown by the dashed lines plotted in Fig. 4g using equations (2) and (3), and the parameters listed in Table 1 (the only adjustment made is for the 4-μm disc, where the calculated *I*_th_ has been reduced by 20% to reproduce asymptotically the experimental slope of *I*_th_ versus *H*). It qualitatively explains the dependence of *I*_th_ at large bias field in both microdiscs, but underestimates its value and fails to reproduce the optimum observed at low bias field.

To understand this behaviour, the finite inhomogeneous contribution to the linewidth Δ*H*_0_ measured in Fig. 2d should be considered as well. In fact, this contribution dominates the full linewidth at low bias field. In that case, the expression of the relaxation rate writes:


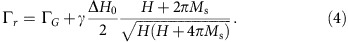


The form of the last term in equation (4) is due to the Kittel dispersion relation and is responsible for the existence of the optimum in *I*_th_ at *H≠*0. Using the value of Δ*H*_0_=0.7 Oe extracted in Fig. 2d for the 2-μm disc in equation (4) in combination with equation (2), the continuous blue line of Fig. 4g is calculated, in very good agreement with the experimental data. To get such an agreement for the 4-μm disc, Δ*H*_0_ has to be increased by 25% compared with the value determined in Fig. 2d. In this case, both the position of the optimum (observed at *H*≃0.3–0.5 kOe) and the exact value of *I*_th_ are also well reproduced for the 4-μm disc, as shown by the continuous red line in Fig. 4g.

Hence, it turns out that quasi-degenerate SW modes, which are responsible for the inhomogeneous contribution to the linewidth, strongly affect the exact value and detailed dependence versus *H* of *I*_th_. In fact, it is the total linewidth that truly quantifies the losses of a magnetic device regardless of the nature and number of microscopic mechanisms involved. Even in structures with micron-sized lateral dimensions, there still exist a few quasi-degenerate SW modes as evidenced by the finite Δ*H*_0_ observed in Fig. 2d. Owing to magnon–magnon scattering, they are linearly coupled to the main FMR mode, which as a result has its effective damping increased, along with the threshold current. The presence of these SW modes is also known to have a crucial role in SOT-driven dynamics. The strongly non-equilibrium distribution of SWs promoted by SOT in combination with nonlinear interactions between modes can lead to mode competition, which might even prevent auto-oscillations to start^33^. We believe that the observed behaviours of the integrated power (Fig. 4d) and linewidth (Fig. 4e) versus *I*_dc_ are reminiscent of the presence of these quasi-degenerate SW modes. A meaningful interpretation of these experimental results is that as the FMR mode starts to auto-oscillate and to grow in amplitude as the d.c. current is increased above the threshold, its coupling to other SW modes—whose amplitudes also grow because of SOT—becomes larger, which makes the flow of energy out of the FMR mode more efficient. This reduces the inductive signal, as non-uniform SW modes are poorly coupled to our inductive detection scheme. At the same time, it enhances the auto-oscillation linewidth, which reflects this additional nonlinear relaxation channel.

The smaller inhomogeneous linewidth in the 2-μm disc (Fig. 2d) results in a field dependence of the threshold current closer to the one expected for the purely homogeneous case (Fig. 4g). This indicates that reducing further the lateral size of the microstructure will allow to completly lift the quasi degeneracy between spin-wave modes^25^, as predicted by micromagnetic simulations, which show that this is obtained for lateral sizes <1 μm. This could extend the stability of the auto-oscillation for the FMR mode, and experimental techniques capable of detecting SWs in nanostructures^26^,^33^ should be used to probe this transition. Very importantly for the field of magnonics, it was recently shown that this constraint on confinement could be relaxed in one dimension such as to produce a propagation stripe^34^. Other strategies might consist in using specific non-uniform SW modes or to engineer the SW spectrum using topological singularities such as vortices or bubbles, which could be most relevant to design active magnonics computational circuits.

## Methods

### Samples

Details of the PLD growth of the YIG layer can be found in ref. 19. Its dynamical properties have been determined by broadband FMR measurements. The transport parameters of the 8-nm-thick Pt layer deposited on top by magnetron sputtering have been determined in a previous study^27^. The YIG|Pt microdiscs are defined by e-beam lithography, as well as the Au(80 nm)|Ti(20 nm) electrodes—separated by 1 μm from each other—which contact them. This electrical circuit is insulated by a 300-nm-thick SiO_2_ layer, and a broadband microwave antenna made of 250-nm-thick Au with a 5-μm-wide constriction is defined on top of each disc by optical lithography.

### Measurements

The samples are mounted between the poles of an electromagnet which can be rotated to vary the angle *φ* shown in Fig. 3a. Two 50 Ω matched picoprobes are used to connect to the microwave antenna and to the electrodes which contact the Pt layer. The latter are connected to a d.c. current source through a bias-tee. To perform ISHE-detected FMR measurements, a microwave synthesizer is connected to the microwave antenna, and the output power is turned on and off at a modulation frequency of 9 kHz. The voltage across Pt is measured by a lock-in after a low-noise preamplifier (gain 100). For the detection of auto-oscillations, high-frequency low-noise amplifiers are used (gain 33–39 dB, depending on the frequency range). Two spectrum analysers simultaneously monitor in the frequency domain the voltages *V*_*x*_ and *V*_*y*_ across the Pt layer and in the microwave antenna, respectively, (Fig. 3a). The resolution bandwidth employed in the measurements is set to 1 MHz.

## Additional information

**How to cite this article**: Collet, M. *et al*. Generation of coherent spin-wave modes in yttrium iron garnet microdiscs by spin–orbit torque. *Nat. Commun*. 7:10377 doi: 10.1038/ncomms10377 (2016).

## Figures and Tables

**Table 1:** Physical parameters of the Pt and bare YIG layers and of the hybrid YIG|Pt bilayer.

**Pt**	***t*_Pt_ (nm)**	***ρ* (μΩ cm)**	***λ*_SD_ (nm)**	**Θ_SH_**
From Rojas-Sánchez *et al*.^27^	8	17.3±0.6	3.4±0.4	0.056±0.010
**YIG**	***t*_YIG_ (nm)**	***α*_0_**	**4*πM*_s_ (G)**	***γ* (10^7^ rad s^−1^ G^−1^)**
This study	20	(4.8±0.5) × 10^−4^	2150±50	1.770±0.005
**YIG|Pt**	***t*_YIG_|*t*_Pt_ (nm)**	***α***	***g*_↑↓_ (10^18^ m^−2^)**	***T***
This study	20|8	(2.05±0.1) × 10^−3^	3.6±0.5	0.2±0.05

*t*_Pt_, thickness; *ρ*, resistivity; *λ*_SD_, spin diffusion length; Θ_SH_, spin Hall angle of the Pt; *t*_YIG_, thickness; *α*_0_, damping parameter; 4*πM*_s_, magnetization; *γ*, gyromagnetic ratio of the YIG; *t*_YIG_|*t*_Pt_, thicknesses; *α*, damping parameter; *g*_↑↓_, spin-mixing conductance; *T*, transparency of the YIG|Pt hybrid.
